# Propensity score-matched comparison of robotic- and video-assisted thoracoscopic surgery, and open lobectomy for non-small cell lung cancer patients aged 75 years or older

**DOI:** 10.3389/fonc.2022.1009298

**Published:** 2022-09-16

**Authors:** Hanbo Pan, Zenan Gu, Yu Tian, Long Jiang, Hongda Zhu, Junwei Ning, Jia Huang, Qingquan Luo

**Affiliations:** Department of Thoracic Surgical Oncology, Shanghai Lung Cancer Center, Shanghai Chest Hospital, Shanghai Jiao Tong University School of Medicine, Shanghai, China

**Keywords:** non-small cell lung cancer, robot-assisted thoracoscopic surgery, video-assisted thoracoscopic surgery, open lobectomy, elderly patients, propensity score-matched study

## Abstract

**Introduction:**

Although robot-assisted thoracoscopic surgery (RATS) has been widely applied in treating non-small cell lung cancer (NSCLC), its advantages remain unclear for very old patients. The present study compared the perioperative outcomes and survival profiles among RATS, video-assisted thoracoscopic surgery (VATS), and open lobectomy (OL), aiming to access the superiority of RATS for NSCLC patients aged ≥75 years.

**Methods:**

Pathological IA-IIIB NSCLC patients aged ≥75 years who underwent RATS, VATS, or OL between June 2015 and June 2021 in Shanghai Chest Hospital were included. Propensity score matching (PSM, 1:1:1 RATS versus VATS versus OL) was based on 10 key prognostic factors. The primary endpoints were perioperative outcomes, and the secondary endpoints were disease-free (DFS), overall (OS), and cancer-specific survival (CS).

**Results:**

A total of 504 cases (126 RATS, 200 VATS, and 178 OL) were enrolled, and PSM led to 97 cases in each group. The results showed that RATS led to: 1) the best surgical-related outcomes including the shortest operation duration (*p <*0.001) and the least blood loss (*p <*0.001); 2) the fastest postoperative recoveries including the shortest ICU stay (*p* = 0.004), chest tube drainage duration (*p <*0.001), and postoperative stay (*p <*0.001), and the most overall costs (*p <*0.001); 3) the lowest incidence of postoperative complications (*p* = 0.002), especially pneumonia (*p <*0.001). There was no difference in the resection margins, reoperation rates, intraoperative blood transfusion, and volume of chest tube drainage among the three groups. Moreover, RATS assessed more N1 (*p* = 0.009) and total (*p* = 0.007) lymph nodes (LNs) than VATS, while the three surgical approaches dissected similar numbers of N1, N2, and total LN stations and led to a comparable incidence of postoperative nodal upstaging. Finally, the three groups possessed comparable DFS, OS, and CS rates. Further subgroup analysis found no difference in DFS or OS among the three groups, and multivariable analysis showed that the surgical approach was not independently correlated with survival profiles.

**Conclusion:**

RATS possessed the superiority in achieving better perioperative outcomes over VATS and OL in very old NSCLC patients, though the three surgical approaches achieved comparable survival outcomes.

## Introduction

Lung cancer is one of the most prevalent and deadly malignancies worldwide, and non-small cell lung cancer (NSCLC) occupies 80-85% of total lung cancer morbidities (1). Optimal surgical treatment is critical for patients with resectable NSCLC to achieve good long-term outcomes and is becoming increasingly important given the implementation of lung cancer screening approaches has contributed to the earlier diagnosis of the malignancy (2). However, with the average age at diagnosis of approximately 70 years, most NSCLC patients are elderly and are frequently associated with comorbidities and poor cardiopulmonary functions, which have created great challenges for surgical treatments (3). More importantly, NSCLC patients aged ≥75 years who represent up to 40% of total NSCLC cases are associated with less surgical frequencies, more preoperative comorbidities, increased postoperative complications, and worse long-term outcomes compared with those aged 65-74 years (4, 5). Therefore, great attention should be attached to identifying well-tolerated and oncological effective surgical approaches for these very old populations.

Although open lobectomy (OL) is still the standard surgical approach for resectable NSCLC, it is associated with considerable postoperative complications and even surgery-related mortalities, especially in elderly patients (6). Thus, minimally invasive surgeries (MISs) which could reduce postoperative complications and shorten postoperative hospital stay, such as video-assisted thoracoscopic surgery (VATS), have been widely adopted (7). Numerous studies have suggested that VATS achieved better perioperative outcomes and similar long-term survival compared to OL for older NSCLC patients (8–10). Nowadays, robotic-assisted thoracoscopic surgery (RATS), an innovative MIS with high-quality visualization and great maneuverability which allows surgeons to perform complex operations with great convenience and precision, has been increasingly applied in treating NSCLC (11). Currently, a few studies evaluated the safety and effectiveness of RATS in NSCLC patients aged 65 years or older, suggesting that RATS reduced postoperative complications and noncancer-specific mortalities than OL, and assessed increased lymph nodes (LNs) than VATS (3, 12, 13). However, merely a few patients aged ≥75 years were included in these studies, and the advantages of RATS specified for this important group of populations remain unknown.

The present study retrospectively investigated the perioperative outcomes and survival profiles of RATS, VATS, and OL in NSCLC patients aged ≥75 years, aiming to assess the superiority of RATS for very old NSCLC cases. Propensity score-matched (PSM) analysis was applied to mitigate the patient selection bias.

## Methods

### Study design

This study was a single-center retrospective cohort study focusing on NSCLC patients aged ≥75 years who underwent lobectomy at the Department of Thoracic Surgical Oncology, Shanghai Chest Hospital. The Institutional Review Board of Shanghai Lung Tumor Clinical Medical Center, Shanghai Chest Hospital, Shanghai Jiao Tong University approved this study (No. KS1735). All procedures conducted on human participants were following the Declaration of Helsinki (as revised in 2013).

### Patient selection and data collection

We retrospectively identified NSCLC patients aged ≥75 years receiving lobectomy from June 2015 to June 2021. Preoperative exams including pulmonary function testing, electrocardiogram, and echocardiography were conducted to ensure the operation tolerance of patients. Distant metastasis was evaluated by using positron emission tomography/CT (PET/CT), bone scintigraphy, and cranial enhanced magnetic resonance imaging (MRI). Contrast-enhanced chest CT imaging was conventionally used to assess the mediastinal and pulmonary lymph nodal involvement, and PET-CT, endobronchial ultrasound trans-bronchial needle aspiration (EBUS-TBNA), and/or mediastinoscopy were further applied when CT scan indicated a short-axis >1 cm of lymph nodes for suitable patients. For a few patients who could not tolerate or rejected the invasive assessments, CT scan and/or PET-CT were applied for the preoperative lymph nodal evaluation. The inclusion criteria included: aged ≥75 years, underwent RATS, VATS, or OL combining with systemic LNs dissection, and pathologically diagnosed NSCLC. The exclusion criteria included: malignancy other than NSCLC, surgical methods other than lobectomy, neoadjuvant therapy, and preoperative distant metastasis. A total of 504 cases were finally included and divided into the RATS, VATS, and OL groups. Following data were recorded: clinicopathological characteristics including age, gender, smoking status, body mass index (BMI), preoperative comorbidities, pulmonary functions [% of predicted forced expiratory volume in 1 s (FEV1%) and % of predicted diffusing capacity for carbon monoxide (DLCO%)], anatomic location, tumor size, histological type, visceral pleural invasion, and pathological T (pT), N (pN), and TNM (pTNM) stage; perioperative outcomes including resection margins, operation duration, conversion rates, blood loss, intraoperative blood transfusion, ICU stay, duration and volume of chest tube drainage, length of postoperative stay, overall costs, and postoperative complications; LNs assessment including the number of total dissected lymph nodes (LNs) and LN stations, number of harvested N1 and N2 LNs and LN stations and postoperative nodal upstaging; survival profiles including 1-, 3-, and 5-year disease-free (DFS), overall (OS), and cancer-specific survival (CS). Among 504 NSCLC patients identified in our database, 418 cases were staged by the 8^th^ edition of the tumor-node-metastasis (TNM) staging system of the International Association for the Study of Lung Cancer. However, the other 86 enrolled cases were staged according to the 7^th^ TNM version in the database, and therefore these patients were all restaged by the 8^th^ TNM version based on their postoperative paraffin pathology reports before the analyses and propensity score matching.

### Surgical procedures

RATS, VATS, and OL were conducted according to the procedures described previously (6, 14, 15). Briefly, patients received general anesthesia with double-lumen tracheal intubation and contralateral single-lung ventilation and underwent radical pulmonary lobectomy combined with systemic pulmonary and mediastinal LNs dissection. RATS was performed using the da Vinci Surgical System (Intuitive Surgical, Sunnyvale, CA, USA). For RATS and VATS, 4 incisions were created without rib-spreading. For OL, patients received a conventional rib-spreading thoracotomy through an incision of about 15 cm.

### Postoperative management and follow-up

After surgery, patients were discharged from the hospital 1-2 days after removing drainage tubes unless there were comorbidities requiring intervention. Follow-up assessments included thoracic CT and brain MRI scans and were conducted every 3-6 months after the surgery during the first 2-year period and once a year afterward. For patients who did not come to the outpatient clinic regularly, telephone follow-up was performed every 1 year until death or June 2022. Patients lost to follow-up were evaluated based on the latest electronic medical records.

### Statistical analysis

We performed the statistical analysis according to the methods published previously (6, 16–18). Variables were expressed using appropriate descriptive statistics, including frequencies and percentages for categorical variables, and mean ± standard deviation (SD) or median and interquartile range (IQR) for continuous variables. Pearson’s chi-square tests or Fisher’s exact tests with Bonferroni *post-hoc* tests were applied to compare categorical variables. For continuous variables, the normality of distribution and homogeneity of variance was analyzed by Kolmogorov-Smirnov tests, and analysis of variance (ANOVA) was performed if a normal distribution and homogeneity of variance were assumed. If not, the Kruskal-Wallis rank sum tests were performed to compare the three groups, followed by Dunn’s multiple comparisons tests to correct for multiple comparisons. Wilcoxon rank sum tests were applied to compare the conversion rates of RATS and VATS. Survival profiles were analyzed by Kaplan-Meier curves log-rank (Mantel-Cox) tests. Factors relevant to DFS and OS were further analyzed using multivariable Cox’s regression model analysis. Statistical analysis was conducted using SPSS version 26.0 (IBM Corporation, Armonk, NY, USA), and survival profiles were analyzed using GraphPad Prism 9 (GraphPad Software Inc., San Diego, CA, USA). The *p* value of less than 0.05 was considered to be statistically significant.

To mitigate potential selection bias, propensity score matching (PSM) was applied to balance baseline confounding features of patients among the three groups using the nearest matching method with a 1:1:1 RATS versus VATS versus OL group ratio. Enrolled patients were matched by the following variables: age, gender, history of smoke, BMI, FEV1%, DLCO%, tumor size, anatomic location, histological type, pT, and pN stage. PSM was conducted using R version 4.1.3 (The R Foundation for Statistical Computing, Vienna, Austria).

## Results

### Clinicopathological characteristics of patients

The baseline clinicopathological characteristics of patients were expressed in **Table 1**. Among the three groups, the OL group had the highest proportion males (OL 64.05% vs RATS 53.97% vs VATS 51.50%, *p* = 0.039), the lowest FEV1% (OL 85.40 ± 16.52 vs RATS 90.23 ± 18.42 vs VATS 89.63 ± 15.94, *p* = 0.022) and DLCO% (OL 86.11 ± 19.98 vs RATS 89.14 ± 18.71 vs VATS 93.19 ± 18.70, *p <*0.001), and the largest tumor size (OL 4.05 ± 2.15 vs RATS 2.58 ± 1.16 vs VATS 2.67 ± 1.29 cm, *p <*0.001). The three groups also differed in the tumor location (*p* = 0.034), histology type (*p <*0.001), pT (*p <*0.001), pN (*p* = 0.009), and pTNM (*p <*0.001) stage. Therefore, PSM was used to balance the baseline characteristics of patients among the three groups. Finally, a total of 291 cases were included. As summarized in **Table 2**, three groups were well balanced with a similar distribution of all included characteristics following the application of PSM.

**Table 1:** Baseline clinicopathological characteristics of unmatched populations.

Characteristic	RATS (N = 126)	VATS (N = 200)	OL (N = 178)	*p* Value
Age (y), mean ± SD	77.18 ± 2.42	76.93 ± 1.85	77.38 ± 2.31	0.216
Gender, n (%)				0.039
Male Female	68 (53.97%) 58 (46.03%)	103 (51.50%) 97 (48.50%)	114 (64.05%) 64 (35.95%)	
Smoking status, n (%)				0.274
Never Former Active	59 (46.83%) 21 (16.67%) 46 (36.51%)	92 (46.00%) 28 (14.00%) 80 (40.00%)	71 (39.89%) 21 (11.79%) 86 (48.32%)	
BMI (kg/m^2^), mean ± SD	23.75 ± 2.95	23.83 ± 3.26	23.51 ± 2.96	0.596
DM, n (%)	19 (15.08%)	34 (17.00%)	26 (14.61%)	0.797
CAD, n (%)	15 (11.90%)	20 (10.00%)	19 (10.67%)	0.863
HP, n (%)	48 (38.10%)	81 (40.50%)	74 (41.57%)	0.828
COPD, n (%)	11 (8.73%)	19 (9.50%)	18 (10.11%)	0.921
FEV1 (% of predicted), mean ± SD	90.23 ± 18.42	89.63 ± 15.94	85.40 ± 16.52	0.022
DLCO (% of predicted), mean ± SD	89.14 ± 18.71	93.19 ± 18.70	86.11 ± 19.98	<0.001
History of malignancy, n (%)	5 (3.97%)	9 (4.50%)	8 (4.49%)	0.969
Tumor location, n (%)				0.034
Right upper lobe Right middle lobe Right lower lobe Left upper lobe Left lower lobe	50 (39.68%) 20 (15.87%) 23 (18.25%) 11 (8.73%) 22 (17.46%)	83 (41.50%) 24 (12.00%) 25 (12.50%) 41 (20.50%) 27 (13.50%)	61 (34.27%) 26 (14.61%) 18 (10.11%) 42 (23.60%) 31 (17.42%)	
Histology type, n (%)				<0.001
AIS/MIA Adenocarcinoma Squamous cell Mixed/large cell/others	9 (7.14%) 90 (71.43%) 16 (12.70%) 11 (8.73%)	10 (5.00%) 147 (73.50%) 32 (16.00%) 11 (5.50%)	0 (0.00%) 91 (51.13%) 69 (38.76%) 18 (10.11%)	
Tumor size (cm), mean ± SD	2.58 ± 1.16	2.67 ± 1.29	4.05 ± 2.15	<0.001
Visceral pleural invasion, n (%)	25 (19.84%)	36 (18.00%)	45 (25.28%)	0.207
Pathological T stage, n (%)				<0.001
pTis pT1 pT2 pT3 pT4	3 (2.38%) 71 (56.35%) 45 (35.71%) 6 (4.76%) 1 (0.79%)	1 (0.50%) 107 (53.50%) 68 (34.00%) 18 (9.00%) 6 (3.00%)	0 (0.00%) 53 (29.78%) 84 (47.19%) 25 (14.04%) 16 (8.99%)	
Pathological N stage, n (%)				0.009
pN0 pN1 pN2	106 (84.13%) 14 (11.11%) 6 (4.76%)	160 (80.00%) 23 (11.50%) 17 (8.50%)	121 (67.98%) 33 (18.54%) 24 (13.48%)	
Pathological TNM stage, n (%)				<0.001
0 IA IB IIA IIB IIIA IIIB	3 (2.38%) 67 (53.18%) 26 (20.63%) 6 (4.76%) 15 (11.90%) 9 (7.14%) 0 (0.00%)	1 (0.50%) 96 (43.00%) 36 (18.00%) 12 (6.00%) 29 (14.50%) 22 (11.00%) 4 (2.00%)	0 (0.00%) 40 (22.47%) 39 (21.91%) 15 (8.43%) 43 (24.16%) 34 (19.10%) 7 (3.93%)	

RATS, robotic-assisted thoracoscopic surgery; VATS, video-assisted thoracoscopic surgery; OL, open lobectomy; SD, standard deviation; BMI, body mass index; DM, diabetes mellitus; CAD, coronary artery disease; HP, hypertension; COPD, chronic obstructive pulmonary disease; FEV1, forced expiratory volume in 1 s; DLCO, diffusing capacity for carbon monoxide; AIS, adenocarcinoma in situ; MIA, minimally invasive adenocarcinoma.

**Table 2:** Baseline clinicopathological characteristics of matched populations.

	RATS (N = 97)	VATS (N = 97)	OL (N = 97)	*p* Value
Age (y), mean ± SD	77.39 ± 2.63	77.12 ± 1.96	77.58 ± 2.51	0.555
Gender, n (%)				0.423
Male Female	60 (61.86%) 37 (38.14%)	61 (62.89%) 36 (37.11%)	68 (70.10%) 29 (29.90%)	
Smoking status, n (%)				0.951
Never Former Active	42 (43.30%) 17 (17.53%) 38 (39.18%)	44 (45.36%) 16 (16.49%) 37 (38.14%)	39 (40.21%) 16 (16.49%) 42 (42.30%)	
BMI (kg/m^2^), mean ± SD	23.92 ± 3.04	23.91 ± 3.36	23.66 ± 3.05	0.892
DM, n (%)	15 (15.46%)	13 (13.40%)	12 (12.37%)	0.816
CAD, n (%)	10 (10.31%)	12 (12.37%)	12 (12.37%)	0.875
HP, n (%)	38 (39.18%)	40 (41.23%)	37 (38.14%)	0.904
COPD, n (%)	8 (8.25%)	7 (7.22%)	7 (7.22%)	0.952
FEV1 (% of predicted), mean ± SD	88.85 ± 18.69	89.48 ± 14.77	88.17 ± 17.36	0.876
DLCO (% of predicted), mean ± SD	88.94 ± 18.32	89.81 ± 15.47	88.68 ± 17.28	0.803
History of malignancy, n (%)	2 (2.06%)	3 (3.09%)	3 (3.09%)	1.000
Tumor location, n (%)				0.959
Right upper lobe Right middle lobe Right lower lobe Left upper lobe Left lower lobe	36 (37.11%) 16 (16.49%) 15 (15.46%) 10 (10.31%) 20 (20.62%)	39 (40.21%) 12 (12.37%) 13 (13.40%) 14 (14.43%) 19 (19.59%)	37 (38.14%) 14 (14.43%) 12 (12.37%) 16 (16.49%) 18 (18.56%)	
Histology type, n (%)				0.840
TIS/MIA Adenocarcinoma Squamous cell Mixed/large cell/others	0 (0.00%) 72 (74.23%) 16 (16.49%) 9 (9.28%)	0 (0.00%) 71 (73.20%) 19 (19.59%) 7 (7.22%)	0 (0.00%) 67 (69.07%) 19 (19.59%) 11 (11.34%)	
Tumor size (cm), mean ± SD	2.85 ± 1.15	2.75 ± 1.35	2.90 ± 1.26	0.693
Visceral pleural invasion, n (%)	21 (21.65%)	20 (20.62%)	26 (26.80%)	0.548
Pathological T stage, n (%)				0.981
pTis pT1 pT2 pT3 pT4	0 (0.00%) 49 (50.52%) 41 (42.27%) 6 (6.19%) 1 (1.03%)	0 (0.00%) 47 (48.45%) 40 (41.24%) 8 (8.25%) 2 (2.06%)	0 (0.00%) 47 (48.45%) 40 (41.24%) 9 (9.28%) 1 (1.03%)	
Pathological N stage, n (%)				0.228
pN0 pN1 pN2	79 (81.44%) 13 (13.40%) 5 (5.15%)	76 (78.35%) 13 (13.40%) 8 (8.25%)	66 (68.04%) 21 (21.65%) 10 (10.31%)	
Pathological TNM stage, n (%)				0.707
0 IA IB IIA IIB IIIA IIIB	0 (0.00%) 46 (47.42%) 22 (22.68%) 6 (6.19%) 14 (14.43%) 9 (9.28%) 0 (0.00%)	0 (0.00%) 41 (42.27%) 21 (21.65%) 6 (6.19%) 18 (18.56%) 10 (10.31%) 1 (1.03%)	0 (0.00%) 36 (37.11%) 19 (19.59%) 4 (4.12%) 26 (26.80%) 11 (11.34%) 1 (1.03%)	

RATS, robotic-assisted thoracoscopic surgery; VATS, video-assisted thoracoscopic surgery; OL, open lobectomy; SD, standard deviation; BMI, body mass index; DM, diabetes mellitus; CAD, coronary artery disease; HP, hypertension; COPD, chronic obstructive pulmonary disease; FEV1, forced expiratory volume in 1 s; DLCO, diffusing capacity for carbon monoxide; AIS, adenocarcinoma in situ; MIA, minimally invasive adenocarcinoma.

### Perioperative outcomes

The perioperative outcomes of enrolled patients were shown in **Table 3**. Patients who underwent RATS were associated with the shortest operation duration (RATS 100.85 ± 29.06 vs VATS 113.75 ± 33.40 vs OL 112.76 ± 22.85 mins, *p <*0.001) and the least blood loss (*p <*0.001). RATS also led to the shortest ICU stay (RATS 0[0-1] vs VATS 1[0-1] vs OL 1[0-1] days, *p* = 0.004), chest tube drainage duration (RATS 4[3-6] vs VATS 5[4-6] vs OL 5[5-7] days, *p <*0.001) and postoperative stay (RATS 5[4-6] vs VATS 5[4-7] vs OL 6[5-8] days, *p <*0.001) among three surgical approaches, and had a similar conversion rate compared with VATS (*p* = 0.184). However, the overall cost in the RATS group was $14838.26 ± 2841.65, which was significantly higher than that in the VATS ($13190.51 ± 2120.18, *p <*0.001) and OL ($13429.58 ± 2582.36, *p <*0.001) group. There was no significant difference in terms of the resection margins (*p* = 0.608), reoperation rates (*p* = 0.543), intraoperative blood transfusion (*p* = 0.377), and volume of chest tube drainage (*p* = 0.061) among the three groups. Moreover, patients in the RATS group had the lowest incidence of postoperative complications (RATS 30.93% vs VATS 41.24% vs OL 55.67%, *p* = 0.002). More importantly, patients who received RATS were associated with a significantly lower incidence of pneumonia than those who received VATS (*p <*0.050) or OL (*p <*0.050). Finally, there was no in-hospital or 30-day mortality in all three groups.

**Table 3:** Perioperative outcomes of matched populations.

Characteristic	RATS (N = 97)	VATS (N = 97)	OL (N = 97)	*p* Value	*RATS vs VATS^b^*	*RATS vs OL^b^*	*VATS vs OL^b^*
Resection margins^a^, n (%)				0.608	>0.050	>0.050	>0.050
R0	89 (91.75%)	88 (90.72%)	84 (86.60%)				
R1	8 (8.25%)	9 (9.28%)	12 (12.37%)				
R2	0 (0.00%)	0 (0.00%)	1 (1.03%)				
Reoperation, n (%)	1 (1.03%)	3 (3.09%)	4 (4.12%)	0.543	>0.050	>0.050	>0.050
Operation duration (min), mean ± SD	100.85 ± 29.06	113.75 ± 33.40	112.76 ± 22.85	<0.001	0.009	<0.001	1.000
Blood loss (mL), n (%)				<0.001	<0.050	<0.050	<0.050
<100	72 (74.23%)	54 (55.67%)	35 (36.08%)				
≥100	25 (25.77%)	43 (44.33%)	62 (63.92%)				
Intraoperative blood transfusion, n (%)	0 (0.00%)	2 (2.06%)	3 (3.09%)	0.377	>0.050	>0.050	>0.050
Conversion to thoracotomy, n (%)	1 (1.03%)	4 (4.12%)	–	0.184	–	–	–
ICU stay (days), median [IQR]	0[0-1]	1[0-1]	1[0-1]	0.004	1.000	0.005	0.036
Chest tube drainage, median [IQR]							
Duration (days)	4[3-6]	5[4-6]	5[5-7]	<0.001	0.106	<0.001	0.095
Volume (mL)	800[580-1020]	820[650-1100]	800[650-1130]	0.061	0.464	0.052	0.459
Postoperative stay (days), median [IQR]	5[4-6]	5[4-7]	6[5-8]	<0.001	0.829	<0.001	0.002
Overall costs (USD$), mean ± SD	14838.26 ± 2841.65	13190.51 ± 2120.18	13429.58 ± 2582.36	<0.001	<0.001	<0.001	1.000
Postoperative complications, n (%)	30 (30.93%)	40 (41.24%)	54 (55.67%)	0.002	>0.050	<0.050	>0.050
Pneumonia requiring antibiotics	7 (7.22%)	21 (21.65%)	34 (35.05%)	<0.001	<0.050	<0.050	>0.050
Acute respiratory distress syndrome	2 (2.06%)	3 (3.09%)	2 (2.06%)	1.000	>0.050	>0.050	>0.050
Pulmonary embolism	0 (0.00%)	0 (0.00%)	2 (2.06%)	0.331	>0.050	>0.050	>0.050
Prolonged air leak >5 days	18 (18.56%)	17 (17.53%)	25 (25.77%)	0.302	>0.050	>0.050	>0.050
Subcutaneous emphysema	12 (12.37%)	10 (10.31%)	13 (13.40%)	0.797	>0.050	>0.050	>0.050
Bronchopleural fistula	0 (0.00%)	2 (2.06%)	2 (2.06%)	0.551	>0.050	>0.050	>0.050
Hemorrhage requiring intervention	0 (0.00%)	3 (3.09%)	3 (3.09%)	0.253	>0.050	>0.050	>0.050
Chylothorax	2 (2.06%)	2 (2.06%)	2 (2.06%)	1.000	>0.050	>0.050	>0.050
Pyothorax	0 (0.00%)	2 (2.06%)	3 (3.09%)	0.377	>0.050	>0.050	>0.050
Chest tube reinsertion	1 (1.03%)	3 (3.09%)	4 (4.12%)	0.543	>0.050	>0.050	>0.050
Atrial fibrillation	2 (2.06%)	2 (2.06%)	3 (3.09%)	1.000	>0.050	>0.050	>0.050
Recurrent laryngeal nerve injury	0 (0.00%)	1 (1.03%)	1 (1.03%)	1.000	>0.050	>0.050	>0.050
Wound infection	1 (1.03%)	2 (2.06%)	4 (4.12%)	0.512	>0.050	>0.050	>0.050
In-hospital mortality	0 (0.00%)	0 (0.00%)	0 (0.00%)	–	–	–	–
30 d mortality	0 (0.00%)	0 (0.00%)	0 (0.00%)	–	–	–	–
Readmission	1 (1.03%)	2 (2.06%)	4 (4.12%)	0.512	>0.050	>0.050	>0.050

a Resection margins: R0, no residual tumor; R1, residual microscopic tumor and/or positive upper paratracheal (#2) LN; R2, residual macroscopic tumor.

b adjusted p value of multiple comparisons between every two groups. RATS, robotic-assisted thoracoscopic surgery; VATS, video-assisted thoracoscopic surgery; OL, open lobectomy; SD, standard deviation; IQR, interquartile range.

### LNs assessment

As expressed in **Table 4**, OL harvested the highest number of N1 (OL 5.79 ± 3.62 vs RATS 5.10 ± 2.40 vs VATS 4.18 ± 2.78, *p <*0.001), N2 (OL 7.74 ± 4.29 vs RATS 6.91 ± 4.50 vs VATS 5.63 ± 3.53, *p <*0.001), and total (OL 13.54 ± 6.05 vs RATS 12.01 ± 5.55 vs VATS 9.81 ± 4.55, *p <*0.001) LNs. Nevertheless, RATS dissected comparable N1 (*p* = 0.730), N2 (*p* = 0.289), and total (*p* = 0.075) LNs than OL. When comparing the two MISs, RATS assessed a higher number of N1 (*p* = 0.009) and total (*p* = 0.007) LNs than VATS, while having no superiority over VATS in assessing N2 LNs (*p* = 0.056). Finally, three surgical approaches dissected similar numbers of N1 (*p* = 0.415), N2 (*p* = 0.298), and total (*p* = 0.124) LN stations, and also led to a comparable incidence of postoperative nodal upstaging (*p* = 0.356).

**Table 4:** LNs assessment of matched populations.

Characteristic	RATS(N = 97)	VATS(N = 97)	OL(N = 97)	*p* Value	*RATS vs VATS^a^*	*RATS vs OL^a^*	*VATS vs OL^a^*
Number of N1 LNs, mean ± SD	5.10 ± 2.40	4.18 ± 2.78	5.79 ± 3.62	<0.001	0.009	0.730	<0.001
Number of N1 LN stations, mean ± SD	2.38 ± 0.86	2.26 ± 0.82	2.40 ± 0.86	0.415	1.000	1.000	0.599
Number of N2 LNs, mean ± SD	6.91 ± 4.50	5.63 ± 3.53	7.74 ± 4.29	<0.001	0.056	0.289	<0.001
Number of N2 LN stations, mean ± SD	3.13 ± 1.34	3.14 ± 1.26	3.35 ± 1.24	0.298	1.000	0.427	0.718
Total number of LNs, mean ± SD	12.01 ± 5.55	9.81 ± 4.55	13.54 ± 6.05	<0.001	0.007	0.075	<0.001
Total number of LN stations, mean ± SD	5.52 ± 1.69	5.39 ± 1.57	5.72 ± 1.77	0.124	1.000	0.347	0.167
Nodal upstaging, n (%) cN0-pN1 cN0-pN2 cN1-pN2	9 (9.28%) 5 (5.15%) 3 (3.09%) 1 (1.03%)	10 (10.31%) 6 (6.19%) 3 (3.09%) 1 (1.03%)	15 (15.46%) 7 (7.22%) 6 (6.19%) 2 (2.06%)	0.356 0.837 0.609 1.000	>0.050 >0.050 >0.050 >0.050	>0.050 >0.050 >0.050 >0.050	>0.050 >0.050 >0.050 >0.050

a Adjusted p value of multiple comparisons between every two groups. LNs, lymph nodes; RATS, robotic-assisted thoracoscopic surgery; VATS, video-assisted thoracoscopic surgery; OL, open lobectomy; SD, standard deviation.

### Survival profiles

**Figure 1:**
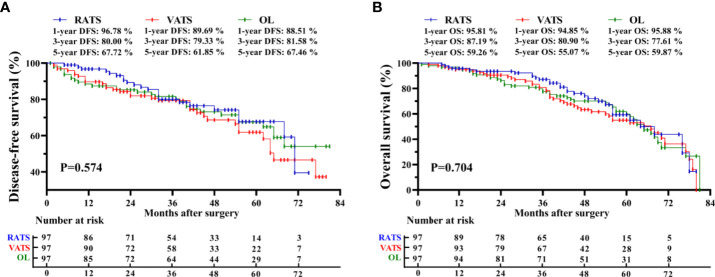
Kaplan-Meier survival curve of matched patients. Comparison of disease-free survival **(A)** and overall survival **(B)** among the RATS, VATS, and OL groups. *RATS, robotic-assisted thoracoscopic surgery; VATS, video-assisted thoracoscopic surgery; OL, open lobectomy*.

The median follow-up of the RATS, VATS, and OL groups was 43[7-80], 44[3-80], and 53[1-81] months, respectively. In the RATS group, 1-, 3-, and 5-year DFS rates were 96.78%, 80.00%, and 67.72%, respectively, and 1-, 3-, and 5-year OS rates were 95.81%, 87.19%, and 59.26%, respectively (**Figure 1**). Besides, patients receiving VATS had the 1-, 3- and 5-year DFS rates of 89.69%, 79.33%, and 61.85%, respectively, and possessed the 1-, 3- and 5-year OS rates of 94.85%, 80.90%, and 55.07%, respectively. Moreover, OL led to the 1-, 3- and 5-year DFS rates of 88.51%, 81.58% and 67.46% respectively, and 1-, 3- and 5- OS rates of 95.88%, 77.61% and 59.87%, respectively. The three groups possessed comparable DFS (*p* = 0.574) and OS (*p* = 0.704). Moreover, the three surgical approaches also achieved similar CS rates (*p* = 0.470, **Supplementary Figure S1**). Further subgroup analyses also suggested no survival profile difference among the three groups in terms of pTNM or pN stage (**Supplementary Figure S2**). Furthermore, we found that the surgical type was not independently correlated with DFS [hazard ratio = 1.190, *p* = 0.478; **Table 5**) or OS (hazard ratio = 1.162, *p* = 0.480) through multivariable Cox regression analysis. Nevertheless, the LNs metastasis was independently correlated with shortened DFS (HR = 3.785, *p <*0.001) and OS (HR = 1.857, *p <*0.001).

**Table 5:** Cox’s proportional hazards regression model analysis for survival profiles of matched populations.

Predictors of survival	DFS			OS		
	*p* Value	HR	95% CI	*p* Value	HR	95% CI
Surgical type (RATS vs others)	0.478	1.190	0.736, 1.923	0.480	1.162	0.766, 1.764
Gender (male vs female)	0.462	0.836	0.518, 1.348	0.885	0.971	0.648, 1.453
Smoking history (yes vs no)	0.821	1.051	0.684, 1.613	0.631	1.093	0.759, 1.535
Histologic type (ADC vs SCC)	0.442	1.192	0.762, 1.864	0.675	1.086	0.738, 1.600
Tumor size (≤3 vs >3 cm)	0.888	0.995	0.928, 1.067	0.626	0.976	0.885, 1.077
LNs metastasis (yes vs no)	<0.001	3.785	2.727, 5.253	<0.001	1.857	1.348, 2.559

DFS, disease-free survival; OS, overall survival; HR, hazard ratio; RATS, robotic-assisted thoracoscopic surgery; ADC, adenocarcinoma; SCC, squamous cell carcinoma.

## Discussion

The robot-assisted surgical system provides surgeons with wide visibilities through high-definition three-dimensional views, improved dexterity by wide-range motioned mechanical wrists, and better maneuverability by delicate instruments, allowing operators to perform complex operations with great convenience and precision (19, 20). Previous studies have shown that RATS led to better perioperative outcomes than OL and harvested more lymph nodes than VATS, and was also associated with the best cost-effective among the three surgical approaches (3, 21–23). Nowadays, the continuing aged population and increased prevalence of NSCLC have contributed to the rapid growth in the number of older people diagnosed with NSCLC (24). Given the increased incidence of preoperative comorbidities and worsening cardiopulmonary functions when individuals grow older, very old patients more frequently experience postoperative complications, slow recoveries, and poor outcomes than younger individuals, which has promoted critical challenges to surgical resections (15, 25). Although the feasibility and oncological efficacy of RATS in younger NSCLC patients have been widely investigated and well established, the research on RATS for very old NSCLC patients is still limited. Our study compared the perioperative outcomes and survival profiles of RATS, VATS, and OL for NSCLC patients aged 75 years or older, suggesting that RATS led to the best surgical-related outcomes, the fastest postoperative recoveries, and the least postoperative complications, especially postoperative pneumonia, among the three surgical approaches, and also assessed more lymph nodes than VATS. Taken together, our results showed for the first time that RATS possesses the superiority in achieving better perioperative outcomes over VATS and OL in very old NSCLC patients.

The most interesting finding of our study was that RATS led to the lowest incidence of postoperative pneumonia, a prevalent postoperative complication that may be a marker of increased long-term mortality in NSCLC patients undergoing surgery, among the three surgical approaches in NSCLC patients aged ≥75 years (26). Such superiority might be partly attributed to the high-definition visualization and improved dexterity and maneuverability provided by the robotic-assisted surgical system which allowed surgeons to perform surgeries more precisely to avoid causing unnecessary damage (19, 20). Besides, RATS also led to shorter surgical duration and fewer blood loss than VATS, which may mitigate the impact of mechanical ventilation and anesthesia and altered internal environments for patients. More importantly, to the best of our knowledge, it was the first time to find that RATS reduced postoperative pneumonia in old NSCLC patients compared with VATS, which might be attributed to the high incidence of this postoperative complication in the very old patients we enrolled which makes this superiority of RATS more apparent.

When considering surgical-related outcomes, RATS reduced intraoperative blood loss compared with VATS and OL. However, VATS had a similar conversion rate to thoracotomy compared with RATS, and all three groups achieved excellent bleeding control with low incidences of intraoperative blood transfusion. For these reasons, all three surgical approaches appear to be safe and effective with regard to bleeding control for elderly NSCLC patients. Moreover, according to previous studies reported by other surgical teams, the operative time is prevalently longer in robot-assisted surgery than that in VATS or OL due to the additional docking time and the impact of a learning curve (27–29). However, our study indicated that RATS was associated with shortened surgical duration than VATS and OL, which might be attributed to the well-organized surgical team and the experienced operators from a high-volume medical center.

The dissection of LNs is of key importance in the surgical resection of NSCLC. Similar to the results reported by previous studies that enrolled younger patients, the total number of LNs harvested by RATS was 12.01 ± 5.55 in our study, suggesting that LNs dissection using robot-assisted surgical systems may not be significantly affected by growth in ages (11, 15, 30, 31). Nowadays, numerous studies have compared RATS with VATS and/or OL in terms of LNs dissection, but have drawn conflicting conclusions. Jin *et al.* and Haruki *et al.* independently reported that RATS harvested more N1 and total LNs than VATS, while other studies indicated that RATS was comparable to VATS with regard to LNs dissection (11, 30). Moreover, by comparing RATS, VATS, and OL, Toker et al. found that RATS dissected more N1 and total LNs than VATS and OL (21). However, Kneuertz et al. reported that RATS, VATS, and OL dissected a similar number of LNs (32). Our study showed that LNs assessed by RATS were comparable to that dissected by OL and more than that harvested by VATS, suggesting that RATS was an effective surgical technic and even superior to VATS regarding LNs assessment in very old NSCLC patients. Although OL harvested the most LNs, the rate of nodal upstaging of the three groups was comparable. This might be explained by most of the enrolled cases had the early-stage disease without nodal involvement and three surgical approaches assessed a similar number of LN stations. The relevance between LNs assessment and long-term survival remains controversial. Dezube et al. and Hennon et al. independently reported that additional LNs dissection conferred no survival benefit for lobectomy, while other studies suggested that increased LNs assessment was associated with better long-term outcomes (33–36). In our study, increased LNs sampling was not correlated with prolonged DFS and OS in very old NSCLC patients undergoing lobectomy, and further follow-up is still necessary to confirm this result.

Although various kinds of preoperative LNs assessment approaches have been prompted, there is still a 3%-15% of rate occult N2 disease identified at the pathological stage (11, 32, 37–40). However, the occult N2 disease could lead to a poor prognosis and therefore influence the survival profiles in our study. In our hospital, mediastinal LNs were systemically assessed by using thoracic CT and PET-CT, and invasive approaches including EBUS-TBNA and mediastinoscopy were further applied when necessary to minimize the incidence of occult N2 disease. Moreover, in our study, the incidence of occult N2 disease in RATS, VATS, and OL groups was 4.12%, 4.12%, and 8.25%, respectively, which was consistent with many previous studies (11, 32, 37–40). Our results also showed that the three groups had comparable incidences of postoperative lymph nodal upstaging. Therefore, the occult N2 disease may not change our survival outcomes.

When considering the oncological effectiveness, our study showed that RATS achieved comparable DFS, OS, and CS as VATS and OL, and further subgroup analysis also indicated similar survival profiles in terms of pTNM and pN stages among three surgical approaches, suggesting that RATS might be an effective surgical method for both early- and advanced-stage resectable NSCLC patients aged ≥75 years. However, our recruitment ended in June 2021 and only a few patients had long-term follow-up data. In our hospital, RATS was performed for the first time in 2009 by our surgical team (which was also the first RAT in China mainland) and widely applied since 2015. The poor surgical tolerances and high surgical risks of very old NSCLC patients have created great challenges for our surgeons, requiring the operators to be experienced and highly skilled, therefore many enrolled patients underwent RATS in recent years. In order to avoid potential bias due to the surgical dates, we included patients who received RATS, VATS, or OL in a similar period. Consequently, long-term follow-up data were available for a few patients. Nevertheless, the median follow-up of the RATS, VATS, and OL groups was 43[7-80], 44[3-80], and 53[1-81] months, respectively, and follow-up of the particular patient was less than 1 year due to his/her death. Therefore, 3-year survival data were available for most patients, and our results suggested that the three groups possessed comparable 1- and 3-year DFS, OS, and CS. More importantly, the primary endpoints of our study were perioperative outcomes and the results showed that RATS possessed the superiority in achieving better perioperative outcomes over VATS and OL in very old NSCLC patients. The DFS, OS, and CS were the secondary endpoints and we are continuing the follow-up and also enrolling more eligible cases currently, aiming to further compare the long-term survival outcomes of RATS, VATS, and OL based on a larger cohort and the longer follow-up data, and the results will be reported afterward. We also noticed that for pathological I stage NSCLC, all three surgical approaches achieved lower 5-year OS than DFS. This was attributed to the fact that a high proportion of elderly patients with early-stage NSCLC died from non-tumor-specific factors, such as cardiovascular diseases, cerebrovascular accidents, and dysfunction of critical organs. Nevertheless, the relapse and metastasis of malignancy was still the major reason contributing to mortalities of II-III stage NSCLC patients in our cohorts.

There are still some limitations of this study. Despite PSM being used, enrolled patients were not randomized before the surgery and the retrospective nature of this study might lead to undiscovered selection bias. Thus, further randomized, controlled trials are necessary to validate the results of our study. Moreover, this study was performed in a single high-volume center, which largely limited the representativeness of participants, thus further multi-center researches are essential to confirm whether the present study could represent real-world practices. Finally, for patients with relapsed disease, the recurrence patterns (locally or distant) and the relevance to surgical approaches were not described, and further studies are needed.

## Conclusion

In summary, we retrospectively compared the perioperative outcomes and survival profiles of RATS, VATS, and OL in treating NSCLC patients aged 75 years or older. The results suggested that RATS possessed the superiority in achieving better perioperative outcomes over VATS and OL in very old NSCLC patients, though the three surgical approaches achieved comparable survival outcomes.

## Data availability statement

The original contributions presented in the study are included in the article/**Supplementary Material**. Further inquiries can be directed to the corresponding authors.

## Ethics statement

The studies involving human participants were reviewed and approved by Institutional Review Board of Shanghai Chest Hospital. Written informed consent for participation was not required for this study in accordance with the national legislation and the institutional requirements.

## Author contributions

JH and QL contributed to the study design. HP, ZG, and YT were responsible for interpreting the results. LJ, HZ, and JN contributed to the statistical analysis. JH and QL wrote the manuscript. All authors contributed to data collection and analysis. All authors contributed to the article and approved the submitted version.

## Funding

This work was supported by the National Nature Science Foundation of China (Grant No. 81972176). The funders were not involved in the study design, collection, analysis, or interpretation of data, the writing of this article, or the decision to submit it for publication.

## Conflict of interest

The authors declare that the research was conducted in the absence of any commercial or financial relationships that could be construed as a potential conflict of interest.

## Publisher’s note

All claims expressed in this article are solely those of the authors and do not necessarily represent those of their affiliated organizations, or those of the publisher, the editors and the reviewers. Any product that may be evaluated in this article, or claim that may be made by its manufacturer, is not guaranteed or endorsed by the publisher.
