# Supplementary material for: Propensity score-matched comparison of robotic- and video-assisted thoracoscopic surgery, and open lobectomy for non-small cell lung cancer patients aged 75 years or older
Source: Front Oncol. 2022 Sep 16;12:1009298. doi: 10.3389/fonc.2022.1009298 (PMC9525021; doi:10.3389/fonc.2022.1009298)
Supplement: Supplementary file 1 [file DataSheet_1.docx]

***Supplementary Materials***


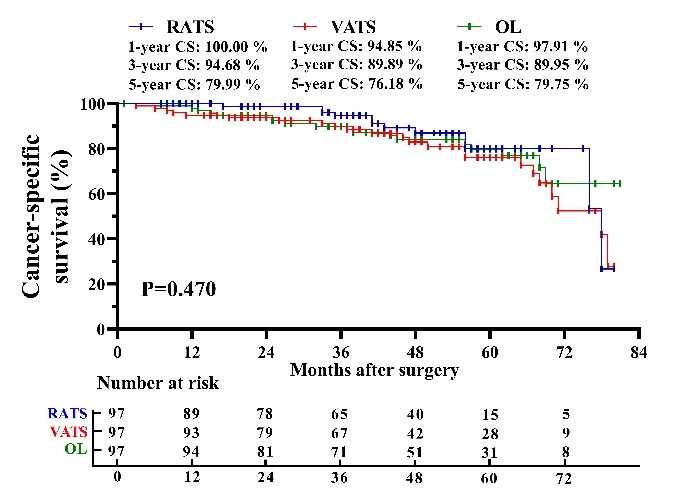


**Supplementary Figure S1.** Comparison of cancer-specific survival among the RATS, VATS, and OL groups. *RATS, robotic-assisted thoracoscopic surgery; VATS, video-assisted thoracoscopic surgery; OL, open lobectomy, CS, cancer-specific survival.*


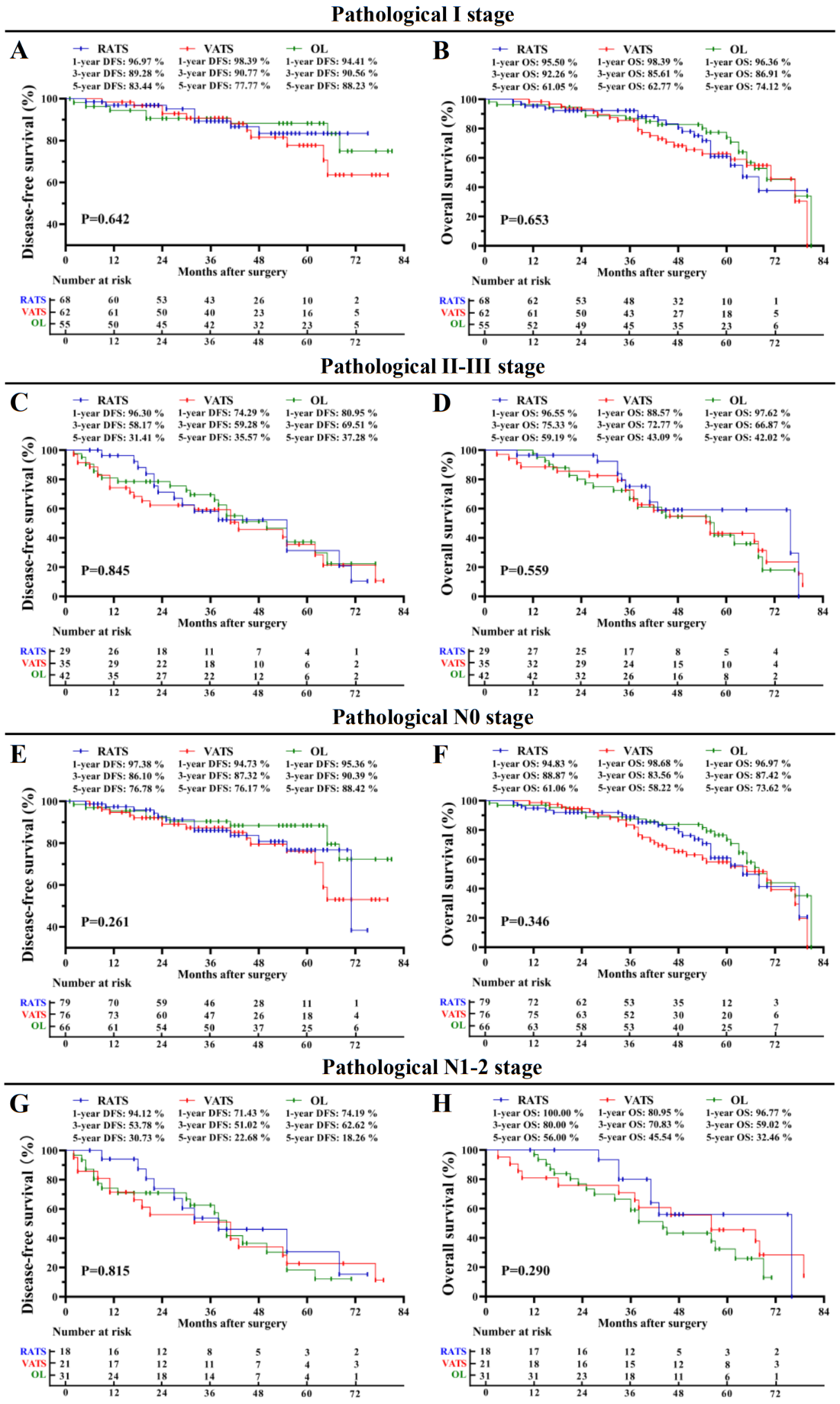


**Supplementary Figure S2.** Subgroup analysis of matched patients. Comparison of DFS (A) and OS (B) among the RATS, VATS, and OL groups in pathological I stage NSCLC. Comparison of DFS (C) and OS (D) among the RATS, VATS, and OL groups in pathological II-III stage NSCLC. Comparison of DFS (E) and OS (F) among the RATS, VATS, and OL groups in pathological N0 NSCLC. Comparison of DFS (G) and OS (H) among the RATS, VATS, and OL groups in pathological N1 NSCLC. *DFS, disease-free survival; OS, overall survival; RATS, robotic-assisted thoracoscopic surgery; VATS, video-assisted thoracoscopic surgery; OL, open lobectomy.*
